# Meloxicam transdermal delivery: effect of eutectic point on the rate and extent of skin permeation

**Published:** 2014-02

**Authors:** Soliman Mohammadi-Samani, Gholamhossein Yousefi, Farhad Mohammadi, Fatemeh Ahmadi

**Affiliations:** 1 Department of Pharmaceutics, School of Pharmacy, Shiraz University of Medical Sciences, Shiraz, Iran; 2 Center for Nanotechnology in Drug Delivery, Shiraz University of Medical Sciences, Shiraz, Iran

**Keywords:** Differential scanning calorimetry, Eutectic mixture, Meloxicam, Skin permeation enhancer, Thymol

## Abstract

***Objective(s):*** Drug delivery through the skin can transfer therapeutic levels of drugs for pharmacological effects. Analgesics such as NSAIDs have gastrointestinal side effects and topical dosage forms of these drugs are mainly preferred, especially for local pains. Meloxicam is one of NSAIDs with no topical form in the market. In this research, we attempted to quantify the skin permeation of a meloxicam topical preparation and to show how permeation would be increased by using thymol as an enhancer. The effect of eutectic point of drug and thymol mixture on rate and extent of skin permeation was also studied.

***Materials and Methods:*** Different mixtures of thymol and meloxicam (2:8, 4:6, 5:5, 6:4, 8:2) were prepared and their melting point were obtained by differential scanning calorimetry. Then drug permeation was measured using diffusion cells and the Guinea pig skin.

***Results:*** Mixtures in ratios 5:5 and 4:6 of meloxicam / thymol showed a new endotherm at 149 and 140°C in DSC thermograms. The permeability of meloxicam from the creams containing 6:4, 5:5 and 4:6 ratios of meloxicam to thymol were 4.71, 15.2, 22.06 µg/cm^2^ respectively. This was significantly different from the cream of pure meloxicam (3.76 µg/cm^2^).

***Conclusion:*** This study set out to determine that thymol plays as a skin permeation enhancer and increases the meloxicam skin absorption and this enhancement is significant at the eutectic point of drug-enhancer mixture.

## Introduction

Patient compliance has always been one of the major concerns in development of pharmaceutical formulations. To overcome the limitations of the systemic administration of drug including oral and parenteral administration, alternative routes such as topical administration are considered. Transdermal drug delivery is one of these patient-friendly methods of drug delivery which provides several advantages over oral and injectable routes (1). These advantages include decreasing the food-drug intera-ction, premature metabolism in the gut wall and liver, avoiding needles and subsequent complications as well as the large surface area available for systemic and controlled drug delivery (2-4). Indeed, by transdermal delivery of drugs such as NSAIDs, risk of gastrointestinal (GI) adverse effects is reduced (5, 6). Meloxicam is one of NSAIDs which is reported to inhibit COX II more selectively than other NSAIDs. It is used as analgesic in treatment of osteoarthritis and rheum-atoid arthritis and also acts as an antipyretic agent (5, 7). Meloxicam is less toxic than similar NSAIDs and more people are tolerant to its side effects; however, the use of drug is still limited by its GI side effects and low aqueous solubility (8-10). Transdermal delivery of meloxicam can also provide steady plasma levels (11). Therefore, it could be an alternative route of admin-istration for decreasing side effects and increasing patient compliance for local and systemic effects. Meloxicam presents lower skin irritation and toxicity than other NSAIDs (5), but the main obstacle for transdermal delivery of this drug is limited permeation through stratum corneum. Different approaches have been applied to modify the skin barrier properties including physical and chemical methods. Physical methods utilize magnetic, ultrasound, electric current or high velocity particles and some other physical energy input to change the permeation of drugs. Nevertheless, in chemical methods, chemical entities are basically involved, which are able to penetrate and disorganize the skin barrier (3, 12). Among different chemical enhancers tested, terpenes, terpenoids and essential oils are the most popular ones for transdermal delivery (3). The effect of these compounds including menthol, thym-ol, cineol, etc has been examined on transdermal delivery of ibuprofen (13), tetracaine (14), testoster-one (15) and zidovudine (16). Studies showed that terpenes exert these effects by changing the high regularity of the lipids of strateum corneum (17) as well as the formation of eutectic mixture with drugs which decreases their melting point and increases their thermodynamic activity in the skin (18). Eute-ctic mixture is a combination of two chemicals with lower melting point than each of the components and the higher transmembrane permeation (19).

Taking all of these into consideration, we aimed to design a formulation of meloxicam for transder-mal delivery using thymol as the penetration enhancer. Differential scanning calorimetry (DSC) was applied to detect interaction of drug with thymol in different ratios. Then the optimum ratio was used for transdermal formulation and the *in vitro* skin permeation of the formulation was assessed using Franz-cell diffusion test.

## Materials and Methods

***Materials***

Meloxicam was supplied by Iran Hormone Pharm Co (Tehran, Iran). Thymol, Tween 80, sodium hydroxide, sodium lauryl sulfate (SLS), acetic acid, chloroform and HPLC grade acetonitrile were purch-ased from Merck chemical company (Darmstadt, Ger-many). Carbopol was obtained from BF Goodrich (USA), liquid paraffin was received from Kian Kaveh pharmaceutical and chemicals industries (Iran). All other chemicals and materials were of analytical grade.

***Preparation of meloxicam-thymol mixtures***

To evaluate the interaction of drug with pene-tration enhancer, the two components should be mixed in molecular level (13). Two methods were applied for preparing meloxicam-thymol mixtures. In the first method, meloxicam and thymol in ratios of 8:2, 6:4, 5:5, 4:6, 2:8 were mixed in solid state by mortar and pestle. Total weight of the mixture was kept constant at 50mg. In the second method, solid mixtures of meloxicam and thymol was made by dissolving the components in 10 ml chloroform and keeping the mixture at room temperature for 24 hr until the solvent was fully evaporated. Then, the resulting solid residue was used as meloxicam thymol mixture.

To prevent thymol volatility, mixing of thymol and meloxicam was performed very slowly and in a room with controlled temperature. Following that, minimum pressure was applied during mixing to prevent heating of the mixture. Formulation of meloxicam-thymol mixture in cream also prevents the volatile content from sublimation.

***Differential scanning calorimetry (DSC)***

DSC studies were performed to determine the thermal behavior of meloxicam, thymol and their interaction upon mixing. DSC thermograms were recorded using DSC apparatus BÄHR Thermoanalyse GmbH, Type 302. Sample (5 mg) was sealed in an aluminum pan and DSC was run against a similar pan containing alumina as the reference material. Then, samples were heated from 20 to 270°C at heating rate 5°C/min. DSC was calibrated by indium (melting point, 156.6 ± 0.2°C) as a standard. All DSC runs were performed in triplicates.

***Preparation of water in oil cream of meloxicam-thymol mixture***

A w/o cream of meloxicam-thymol was prepa-red. Aqueous phase consisted of tween 80, distilled water and carbopol and oil phase contained liquid paraffin and meloxicam-thymol mixtures. The aque-ous phase was added into the oil phase and mixed by turbine mixer and NaOH solution 0.2 N was added to provide the required consistency and viscosity by neutralizing carbopol. Details of cream formulations are presented in Table 1.

***In vitro skin permeation test***

Guinea pig abdominal skin was used for skin permeation test. Guinea pigs were euthanized with high dose of ether inhalation and the abdominal skin hair was shaved and the skin was cut and detached using a surgical knife. Skin was then fixed between the donor and acceptor compartment of a home-made Franz diffusion cell. Three cells with capacity 40 ml and diffusion area 5.72 cm^2^ were connected serially to circulation pump to maintain the temper-ature of the diffusion cells at 34°C and receptor phase consisted of 1% SLS solution in water to pre-serve sink condition. For determining the solubility of meloxicam in receptor phase, a supersaturated solution of meloxicam in SLS solution was prepared. For this, excess amount of meloxicam was added to SLS solution on the stirrer and was stirred until no change in concentration determined by HPLC was observed. Then the mixture was filtered and drug solubility in SLS was measured by HPLC. This experiment was repeated three times.

**Table 1:** Composition of cream formulations prepared from different ratios of thymol-meloxicam

Cream formulations	Amount of ingredients (g)					
	Meloxicam	Thymol	Tween 80	Carbopol	Liquid paraffin	Water
Thymol-meloxicam (8:2)	0.2	0.8	0.2	0.1	4	16
Thymol-meloxicam (6:4)	0.2	0.3	0.2	0.1	4	16
Thymol-meloxicam (5:5)	0.2	0.2	0.2	0.1	4	16
Thymol-meloxicam (4:6)	0.2	0.13	0.2	0.1	4	16
Thymol-meloxicam (2:8)	0.2	0.05	0.2	0.1	4	16
Meloxicam	0.2	-	0.2	0.1	4	16

**Figure 1:**
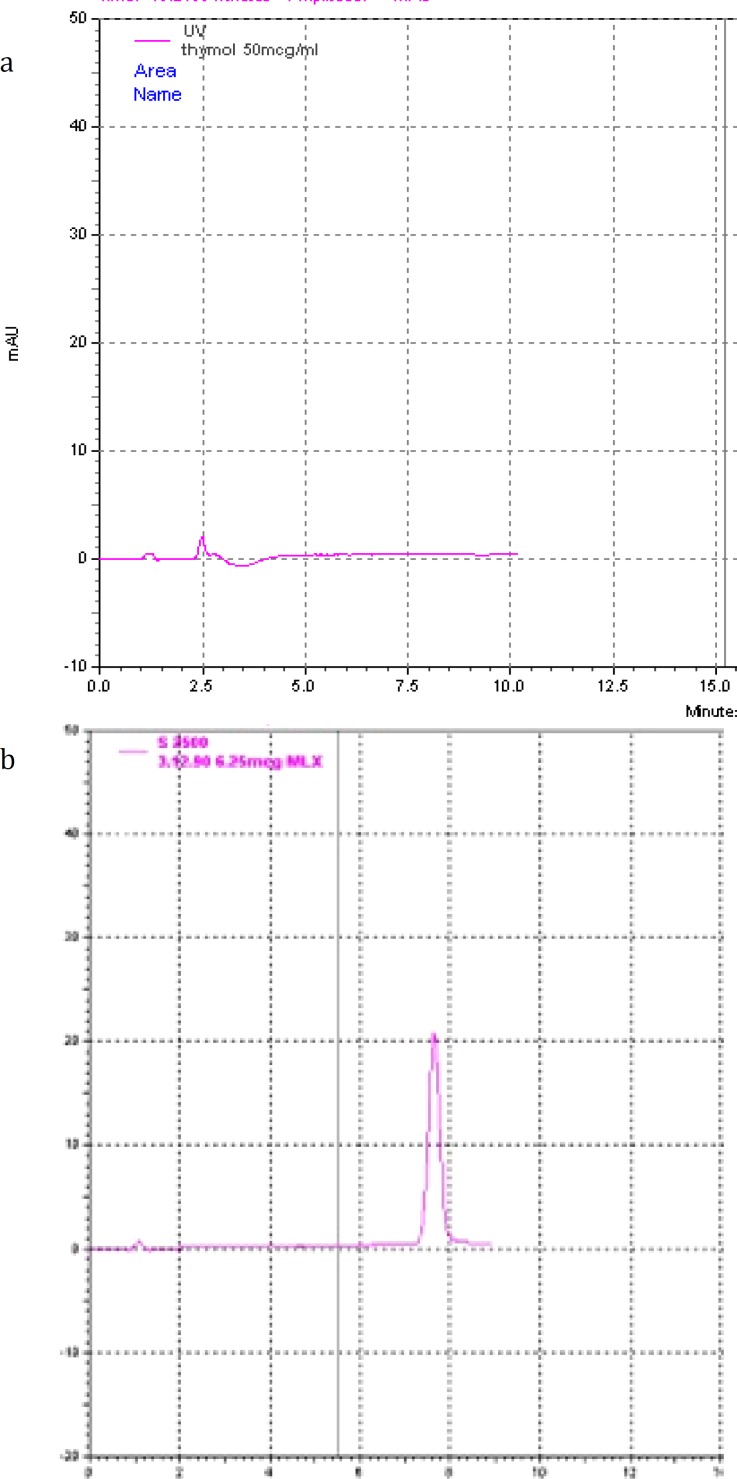
Representative chromatograms of solutions of a) thymol (50 µg/ml) and b) meloxicam (6.25 µg/ml) in acetonitrile

After temperature equilibrium, 200 mg samples of the test cream were rubbed on the epidermal surface of the fixed skin. Five ml samples of the receptor phase were withdrawn at 15, 30, 60, 120, 240, 360 and 480 min and aliquot amounts of fresh buffer was substituted. The samples were kept in -20^°^C freezer and the concentration of the drug in the samples were analyzed using a Knauer HPLC.

***High performance liquid chromatography (HPLC)***

Determination of meloxicam was performed by HPLC. The chromatographic system used was Knauer (Smartline®, Model 1000, Germany) equipped with UV detector. Data was integrated using Eurochrome software for HPLC (Knauer, Germany). The HPLC method was developed according to previous studies with some modifications (20-22). Mobile phase was consisted of acetonitrile: water 50:50 (pH 3) which pumped to the column at flow rate 1.2 ml/min. A C_18_ column (150 mm×4.6 mm) was used and detection wavelength was set to 355 nm. Mobile phase was prepared freshly every day and degassed using vacu-um filtration and ultrasonication.

**Table 2:** Melting points of meloxicam and thymol in mixtures determined by DSC

Chemical composition	Melting points °C (T onset)	
	Mixing in dry state	Mixing in solution
Thymol	48.9	-
Thymol-meloxicam (8:2)	246.7	232.5
Thymol-meloxicam (6:4)	140.2	225
Thymol-meloxicam (5:5)	149, 211.9	209.9
Thymol-meloxicam (4:6)	223	211.6
Thymol-meloxicam (2:8)	210.9	219.9
Meloxicam	240.9	-

Linearity of the method was tested by constru-cting calibration curve of the standard solutions in the concentration range 0.01-50 µg/ml. Reproducibi-lity and precision of the method was also examined by calculating inter- and intra-day variations.

## Results

***HPLC analysis***

The developed HPLC method was linear in the concentration range 0.01-50 µg/ml and the obtained regression factor (R^2^) was 0.9989. The peaks of meloxicam and thymol were completely separated by the developed method without any interference (retention times of thymol and meloxicam were 2.5 and 7.5 min, respectively). Precision of the method was evaluated by repeated injections of the known concentrations of the drug and also calculating the inter- and intra-day variations. The results justify that for all concentrations injected to the system, the variation was less than 10%. Representative chroma-tograms of meloxicam and thymol are depicted in Figure 1.

***DSC studies***

**Figure 2:**
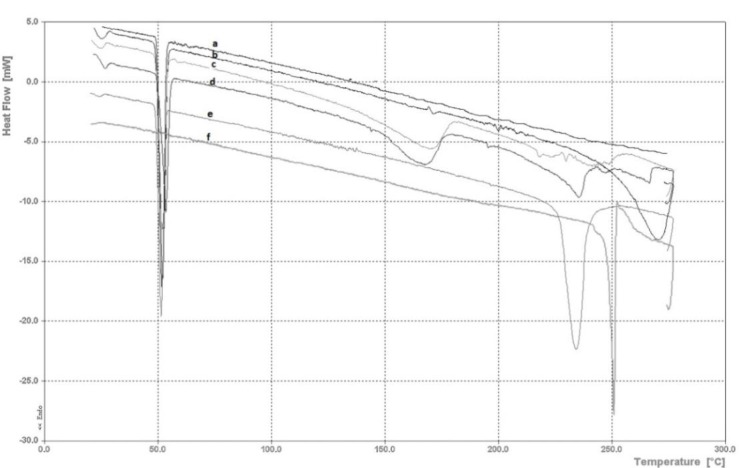
DSC thermograms of a) pure thymol, b) thymol-meloxicam 8:2, c) thymol-meloxicam 6:4, d) thymol-meloxicam 5:5, e) thymol-meloxicam 4:6 and f) pure meloxicam

Meloxicam and thymol melting point was found to be at 240.9 and 48.9 °C, respectively. By increasing the ratio of thymol to meloxicam in mixtures, the melting point of the drug was decreased. There was not a significant difference in the melting points between two mixing methods. Mixtures containing 2:8 and 4:6 ratios of meloxicam to thymol presented lowest melting point. DSC thermograms of the pure drug and the binary mixtures are shown in Figure 2 and the data are summarized in Table 2.

Three ratios 4:6, 5:5 and 6:4 of meloxicam-thymol which presented lower melting points and seems to provide higher skin permeation were selected for skin permeation tests.

***Skin permeation test***

Meloxicam is practically insoluble in water and its aqueous solubility has been reported to be 8 μg/ml (23). To preserve the sink condition in the receptor phase of diffusion cell, 1% solution of SLS in water was used as the receptor phase and the solubility of meloxicam in this medium was 46 μg/ml. 

**Figure 3:**
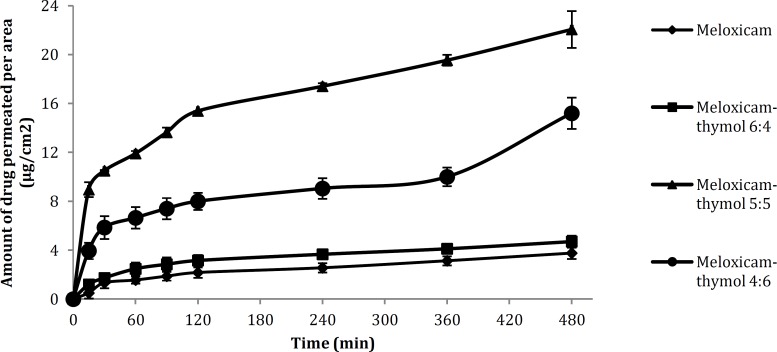
Permeation profile of the drug from the creams prepared by drug alone and different drug-thymol mixtures

Results of skin permeation of the cream contained 1% meloxicam showed that the net amount of drug permeated through Guinea pig skin per area of diffusion was 3.76 μg/cm^2^. Three different cream formulations were prepared from thymol-meloxicam mixtures in the ratio 4:6, 5:5 and 6:4 which were showing the higher melting point depression among the other ratios of meloxicam: thymol and were examined in skin permeation test. The permeation profiles of the drug in the creams prepared from drug alone and drug-thymol mixtures are presented in Figure 3. Results showed that by mixing the drug with thymol, the net amount of drug permeated through the skin was increased significantly compared with the drug alone. Net amount of permeation per area of the diffusion was 4.71 µg/cm^2^, 15.2 µg/cm^2^ and 22.06 µg/cm^2^ for 6:4, 5:5 and 4:6 ratios of meloxicam to thymol. The extent of this permeation enhancement was dependent on the amount of the absorption enhancer in the mix-ture and the melting point depression of the drug.

**Figure 4:**
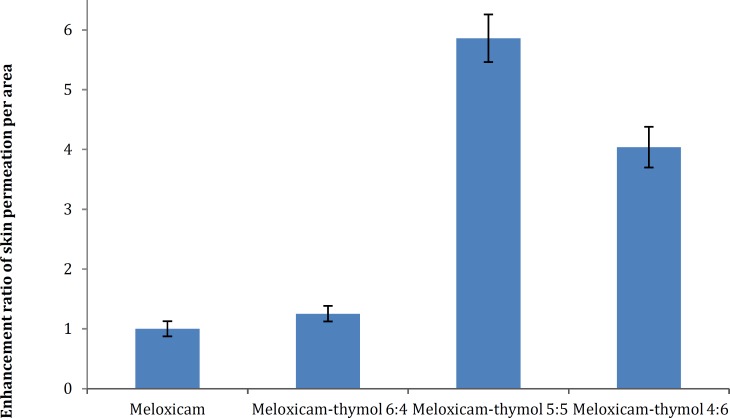
Histogram of enhancement ratio of transdermal permeation of creams containing meloxicam in mixtures with thymol compared with meloxicam alone (calculated from ratio of net flux of each formulation to the formulation containing meloxicam alone

A histogram in Figure 4 represents the enhance-ment ratio of transdermal permeation of meloxicam per diffusion area in different ratios to thymol comp-ared with the cream contained only meloxicam. The presence of thymol also decreased the lag time of permeation of drug and in ratio 5:5 of meloxicam to thymol, the extent of permeation at the first sampling time was 5-times higher than the drug alone. Figure states the effect of thymol as skin absorption enhancer in these formulations.

## Discussion

Meloxicam is a non-steroidal anti-inflammatory drug (NSAID) that is used in a wide range of inflammatory diseases. Side effects of meloxicam limit the use of drug and designing topical formulations can limit meloxicam adverse effects. Meloxicam and thymol are lipophilic materials and have proper solubility in non-polar solvents. Therefore, in the present study it was decided to use oil in water cream with minimum portion of oil phase to maximize the loading of lipophilic drugs in the cream as well as decreasing the risk of retention of drug in the oil phase. For designing the receptor phase for skin permeation test, the low aqueous solubility of meloxicam was a limiting factor. The skin permeation of meloxicam may be enhanced with enhancers that change skin lipids regularity and conformation. Moreover, mixing these chemicals such as terpenoids and essential oils with drugs provide opportunity of formation of eutectic mixture and lowering melting point (3, 17). Among different terpenoids, thymol was used as the permeation enhancer, as there are some reports on the efficacy of this terpene for increasing transdermal delivery along with the use of thymol as an ingredient in topical lotions, creams and solutions. In another study on percutaneous absorption of tamoxifen, it was reported that thymol can increase the partitioning of drug in strateum corneum (24). It is also a lipophilic terpene (Log P 3.28±0.20) which is a good option for increasing permeation of lipophilic drugs such as meloxicam (25). Eutectic mixture is a combination of two or more chemicals which forms a composition with lower melting point (26).

According to DSC data in Figure 2 and Table 2, thymol is melted at 48-49°C and this melting endotherm is not displaced significantly in any of the mixtures with meloxicam. Meloxicam in pure form is melted at 241°C, but by adding thymol to meloxicam, melting point gradually decreased to 238, 223, 212, 223 and 211°C for meloxical-thymol ratios of 8:2, 6:4, 5:5, 4:6 and 2:8, respectively. The interesting point in DSC thermograms, is new endothermic peaks at 149 and 140°C which were observed in DSC results of mixtures containing ratios 5:5 and 4:6 of meloxicam to thymol. This pattern is very similar to eutectic composition with excess of one of the chemicals in the mixture (here thymol) (13, 27). The ratio 5:5 is very near to the eutectic point, with almost complete elimination of the melting endoth-erm at 223°C. There was no considerable difference between mixing meloxicam and thymol in dry state and in solution, just for the intensity of thymol melting endotherm, which is probably related to the evaporation of highly volatile thymol. 

Net amount of drug permeated through Guinea pig skin per area of diffusion from the cream contained 1% meloxicam was 3.76 μg/cm^2^. This extent of permeation was much higher than meloxic-am suspension and was almost similar to meloxicam-loaded transferosomes studied by Duangjit *et al* (8). This basic amount of permeation is related to the solubility of meloxicam in oil phase and emulsifier of the cream and permeation through the lipids of the skin. Relatively low permeation of the drug resulting from this formulation is related to the oily nature of the formulation and viscosity of the vehicle which

reduces the diffusion rate of the drug (28). High solubility in oil phase which results from high value of partition coefficient of meloxicam could also decrease the chemical potential of the drug (29).

As indicated in DSC thermograms in Figure 2, melting point of ratio 6:4 of meloxicam to thymol was lower than pure meloxicam, but for ratios 5:5 and 4:6 a new endothermic peak was observed which is due to the new composition of the eutectic mixture. In addition, a 4-5 fold increase in the skin permeation of these creams was observed. Similar data has been reported on the skin permeation of ibuprofen-thymol mixture (13). It is evident that by increasing the amount of thymol, drug permeation was increased until the ratio of 5:5 is achieved. Following that, increasing the ratio of thymol to 6 presented negative effect on drug permeation. When increasing the amount of thymol, meloxicam is decreasing and the drug is mainly in liquid form. Therefore, amount of solid is decreased. Disappearance of the solid drug in the mixture and therefore lower thermodynamic activity and driving force for permeation is the reason of lower permeation of this ratio (6:4, thymol to meloxicam) compared to the 5:5 ratio. Similar results have been observed in study on skin permeation of thymol-ibuprofen mixtures (13). It was shown in their study that pretreatment with thymol increased the permeation of a saturated solution of ibuprofen by two folds; however, the eutectic mixture provided a much higher increase in skin permeation. Prior studies proved that decrease in melting point of eutectic mixture is the reason of higher permeability of these mixtures in lipid structure of skin. It has been stated for ibuprofen-menthol and lidocaine-prilocaine mixtures that the main reason of enhancement in absorption is increasing lipid solubility of the mixture (13, 30, 31). Certainly, the effect of other factors like excipients of formulations for example surfactants or volatile solvents should not be ignored because these factors could also affect the integrity of the skin structure. Volatile solvent can even influence eutectic point of mixtures. SLS has important effects on integrity of the cell membrane and can therefore change the results of permeation, however, study of effect of 1% SLS on skin penetration of diazepam has shown that this concentration is not effective for changing the skin permeation of the drug (32). In order to limit the effect of these factors, in this study it was tried to keep the composition of all formulations the same. Furthermore, the only determining variable was the ratio of drug to permeation enhancer. Other explanations that may be considered for enhancement of skin permeation are ability of terpenes to fluidize the lipids of the membrane and disorganize them (33), higher distribution of eutectic mixtures in cellular membrane because of lower melting point and higher thermodynamic activity (34), higher access of molecules of eutectic mixture to the membrane (30) and hydrogen bonding between thymol and meloxicam that make them more lipophilic (17).

## Conclusion

One of the significant findings to emerge from this study was that the rate and extent of skin permeation of cream containing 5:5 ratio of meloxicam to thymol was highest amongst all other ratios. This is resulted from higher amount of permeation enhancer, thymol together with the lower melting point of the mixture as stated by DSC studies and higher thermodynamic activity near the eutectic point.
